# First report of four rare strongylid species infecting endangered Przewalski’s horses (*Equus ferus przewalskii*) in Xinjiang, China

**DOI:** 10.1186/s13071-023-05993-w

**Published:** 2023-10-25

**Authors:** Huiping Jia, Sijia Gao, Liping Tang, Yajun Fu, Yu Xiong, Make Ente, Shalitanati Mubalake, Changliang Shao, Kai Li, Defu Hu, Dong Zhang

**Affiliations:** 1 School of Nature Conservation, Beijing Forestry University, Beijing, 100083 China; 2 Xinjiang Research Centre for Breeding Przewalski’s Horse, Xinjiang, China; 3 Xinjiang Kalamaili Mountain Ungulate Nature Reserve Management Center, Xinjiang, China

**Keywords:** *Oesophagodontus robustus*, *Bidentostomum ivashkini*, *Skrjabinodentus caragandicus*, *Petrovinema skrjabini*, Nematodes, Intestinal parasites

## Abstract

**Background:**

The Przewalski's horse (*Equus ferus przewalskii*) is the only surviving wild horse species in the world. A significant population of Przewalski's horses resides in Xinjiang, China. Parasitosis poses a considerable threat to the conservation of this endangered species. Yet, there is limited information on the nematode parasites that infect these species. To deepen our understanding of parasitic fauna affecting wild horses, we identified the intestinal nematodes of Przewalski’s horses in Xinjiang and added new barcode sequences to a public database.

**Methods:**

Between 2018 and 2021, nematodes were collected from 104 dewormed Przewalski's horses in Xinjiang. Each nematode was morphologically identified to the species level, and selected species underwent DNA extraction. The extracted DNA was used for molecular identification through the internal transcribed spacer 2 (ITS2) genetic marker.

**Results:**

A total of 3758 strongylids were identified. To the best of our knowledge, this is the first study to identify four specific parasitic nematodes (*Oesophagodontus robustus*, *Bidentostomum ivashkini*, *Skrjabinodentus caragandicus*, *Petrovinema skrjabini*) and to obtain the ITS2 genetic marker for *P. skrjabini*.

**Conclusions:**

The ITS2 genetic marker for *P. skrjabini* enriches our understanding of the genetic characteristics of this species and expands the body of knowledge on parasitic nematodes. Our findings extend the known host range of four strongylid species, thereby improving our understanding of the relationship between Przewalski’s horses and strongylids. This, in turn, aids in the enhanced conservation of this endangered species. This study introduces new instances of parasitic infections in wild animals and offers the DNA sequence of *P. skrjabini* as a valuable resource for molecular techniques in nematode diagnosis among wildlife.

**Graphical Abstract:**

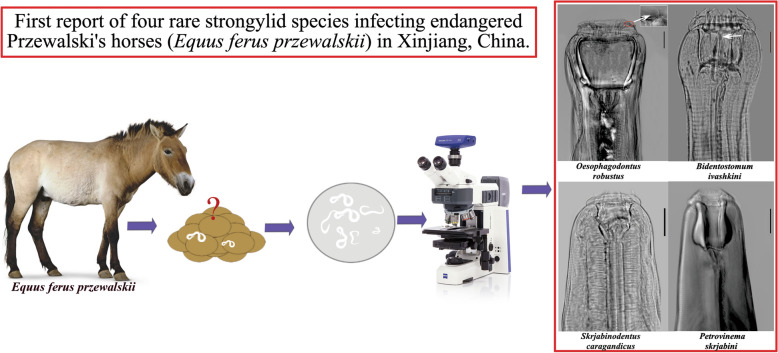

**Supplementary Information:**

The online version contains supplementary material available at 10.1186/s13071-023-05993-w.

## Background

Przewalski's horse (*Equus ferus przewalskii*, PH), classified under the genus *Equus*, is designated as "Endangered" on the IUCN Red List of Threatened Species [1]. In 1969, PHs were declared extinct in the wild [2]. Since 1985, efforts have been made to reintroduce this species into its native habitats in China (Xinjiang and Gansu Provinces) and Mongolia (Takhin Tal Nature Reserve, Hustai National Park and Khomiin Tal) [3]. Given that PHs are endangered, their conservation has become a critical issue. Parasitic infections pose a considerable risk to wildlife conservation [4–7]. While most current research on equids has centered on parasitic infections in domestic horses [8–14], the status of parasitic species and infections in PHs remains inadequately understood [15–19]. Nematodes from the family Strongylidae are predominant in the parasitic community of equids, with a prevalence rate of 100% [17–20].

Globally, 64 species from 19 genera of the Strongylidae family infect equids, with 54 species from 17 genera affecting domestic horses. In contrast, only 38 species have been identified in PHs [8, 15, 21], primarily located in Ukraine, Mongolia and Russia [15–21]. Despite Xinjiang hosting the world's largest population of PHs, only six strongylid species have been recorded in China: *Strongylus vulgaris*, *S. edentatus*, *S. equinus*, *Triodontophorus serratus*, *Cylicocyclus nassatus* and *C. elongatus*[22, 23]. Detection and identification of nematode parasites can be particularly challenging in wildlife settings [6, 24], suggesting that other nematode species may have been overlooked.

In Xinjiang, we implemented regular parasite control measures for PHs and collected parasite samples. The objectives of our study were: (i) to investigate the presence of previously unrecognized parasitic nematode species in the gastrointestinal tract of Przewalski's horses and (2) to augment the reference data for the molecular identification of parasitic strongylids.

## Methods

### Sample collection

The research took place from 2018 to 2021 at two locations in China: the Xinjiang Research Centre for Breeding Przewalski's Horse (XRCBPH: 88.442E, 44.121N) and the Kalamaili Nature Reserve (KNR: 88.594E, 45.141N). Xinjiang Research Centre for Breeding Przewalski's Horse, located in Jimusaer County, is Asia's largest breeding base for PHs. According to statistical data, as of July 2019, Xinjiang was home to 416 surviving PHs, making it the Chinese province with the largest PH population [25].

During each winter of the study period, 7 to 14 PHs were individually dewormed with ivermectin in separate enclosures. Captive PHs received identical treatment. Fecal samples (≥ 200 g of feces/sample) containing all expelled parasites were collected from each horse at KNR at intervals of 18, 24, 42, 48 and 66 h post-treatment. In the XRCBPH study, due to limited enclosure availability, parasite samples were collected based on enclosures, hosting 2 to 12 horses each.

After expulsion in fecal samples, all nematodes were manually collected and washed with a saline solution. These nematodes were subsequently fixed in 70% ethanol and stored at − 20 °C for further identification. Specimens were maintained in the Laboratory of Non-invasive Animals at Beijing Forestry University, Beijing, China.

All sample procedures for this study were carried out with the assistance of a local veterinarian and received approval from the Xinjiang Research Centre for Breeding Przewalski’s Horse (Forestry and Grassland Administration of Xinjiang) and the School of Nature Conservation (Beijing Forestry University).

### Morphological identification

We performed morphological identification on nematodes obtained from 93 captive and 11 wild PHs. The lactophenol clarification procedure was conducted as previously described [26]. All strongylids were classified based on the morphological characteristics outlined by Lichtenfels et al. [8] and Zhang and Kong [10]. Identification was carried out using a ZEISS Axioscope5 upright fluorescence microscope and differential interference-contrast microscopy (both from ZEISS, Germany). Drawings were created using a Zeiss microscope attachment. Photomicrographs were captured with a digital camera, and halftone plates were prepared using Adobe Photoshop.

### Molecular identification

Genomic DNA was extracted from two individuals of each species, specifically *Oesophagodontus robustus*, *Bidentostomum ivashkini*, *Skrjabinodentus caragandicus caragandicus* and *Petrovinema skrjabini skrjabini*, using the TIANamp Micro DNA Kit (Tiangen, China) according to the manufacturer’s instructions. The extracted DNA was stored at – 20 °C. Internal Transcribed Spacer 2 (ITS2) [27] nuclear gene sequences were amplified using specific primers. For PCR amplification, the reaction mixture included 1 μl DNA, 7 μl ddH_2_O, 0.5 μl each ITS2 bidirectional primer (10 μmol/l) and 10 μl 2 × Es Taq MasterMix (Dye) (Beijing Cowin Bioscience Co., Ltd., China). A negative control group was included without a template. The PCR procedure was conducted with a total volume of 20 μl per reaction. The mixture underwent initial denaturation at 94 °C for 3 min, followed by denaturation at 94 °C for 30 s, annealing at 54 °C for 30 s and extension at 72 °C for 45 s. This process was repeated for 35 cycles. A final extension at 72 °C for 10 min was performed. Post-amplification, 3 μl of the PCR products was used for electrophoresis on a 1% agarose gel stained with GoldView. The positively amplified product was purified and sent to the Beijing Genomics Institute (BGI, China) for bidirectional sequencing, as described by Zhang et al. [28]. All obtained sequences were compared with those available in the GenBank database using the Basic Local Alignment Search Tool (BLAST) (http://www.ncbi.nlm.nih.gov/BLAST/).

## Results

In a survey conducted at XRCBPH, 93 PHs from 11 enclosures were tested, resulting in the collection of 1454 nematodes. In a parallel survey by KNR, 11 PHs were tested, yielding 2304 nematodes. Strongylids were identified in all enclosures examined at XRCBPH, showing a 100% infection rate. Similarly, strongylids were detected in every individual tested at KNR, also indicating a 100% infection rate. Parasitic strongylids were found in all tested PHs, with the average intensity of strongylid infection being 15.63 in XRCBPH and 209.45 in KNR.

We identified 23 species of strongylids (Additional file 1: Table S1), among which *Cyathostomum catinatum*, *Cylicostephanus longibursatus* and *Coronocyclus coronatus* had the highest burden. Within this set of 23 species, four specific strongylids—*O. robustus*, *B. ivashkini*, *S. caragandicus* and *P. skrjabini*—were discovered in the PHs. These four species were collected from both XRCBPH and KNR in China (Table 1) and identified based on their morphological characteristics as described by Lichtenfels et al. [8] and Zhang and Kong [10].

**Table 1:** Number of specimens of four species of nematodes (*Oesophagodontus robustus, Bidentostomum ivashkini, Skrjabinodentus caragandicus, Petrovinema skrjabini*) found in Xinjiang Uygur Autonomous Region wild horse Breeding Research Center (XRBRPH) and Kalamaili Nature Reserve (KNR) in China

Locality	Host inspected	Total of *O. robustus* collected	Total of *B. ivashkini* collected	Total of *S. caragandicus* collected	Total of *P. skrjabini* collected
XRBRPH	93	20	1	13	1
KNR	11	1	9	11	33

**Fig. 1:**
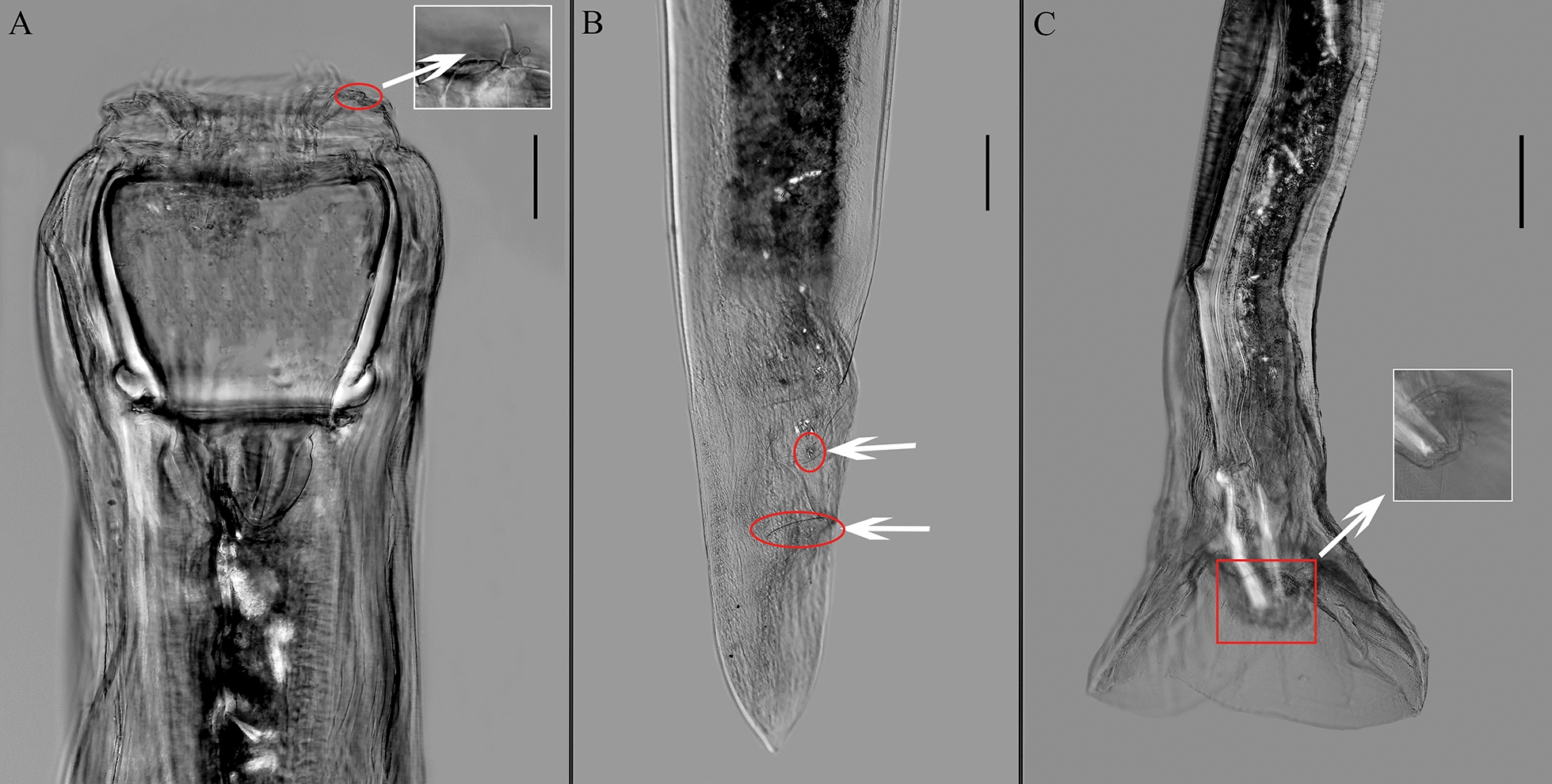
Differential interference micrographs of *Oesophagodontus robustus*. **A** Anterior part of the body, lateral view. Arrow marks submedian papillae, with short bilobed process on stalk and long slender tip and lateral papilla (scale bar: 100 μm). **B** Tail of female. Arrow at the anterior tip marks vulva. Arrow at the posterior tip marks anus (scale bar: 200 μm). **C** Male tail, lateral view. Genital cone, lateral view (arrow) (scale bar: 200 μm)

**Fig. 2:**
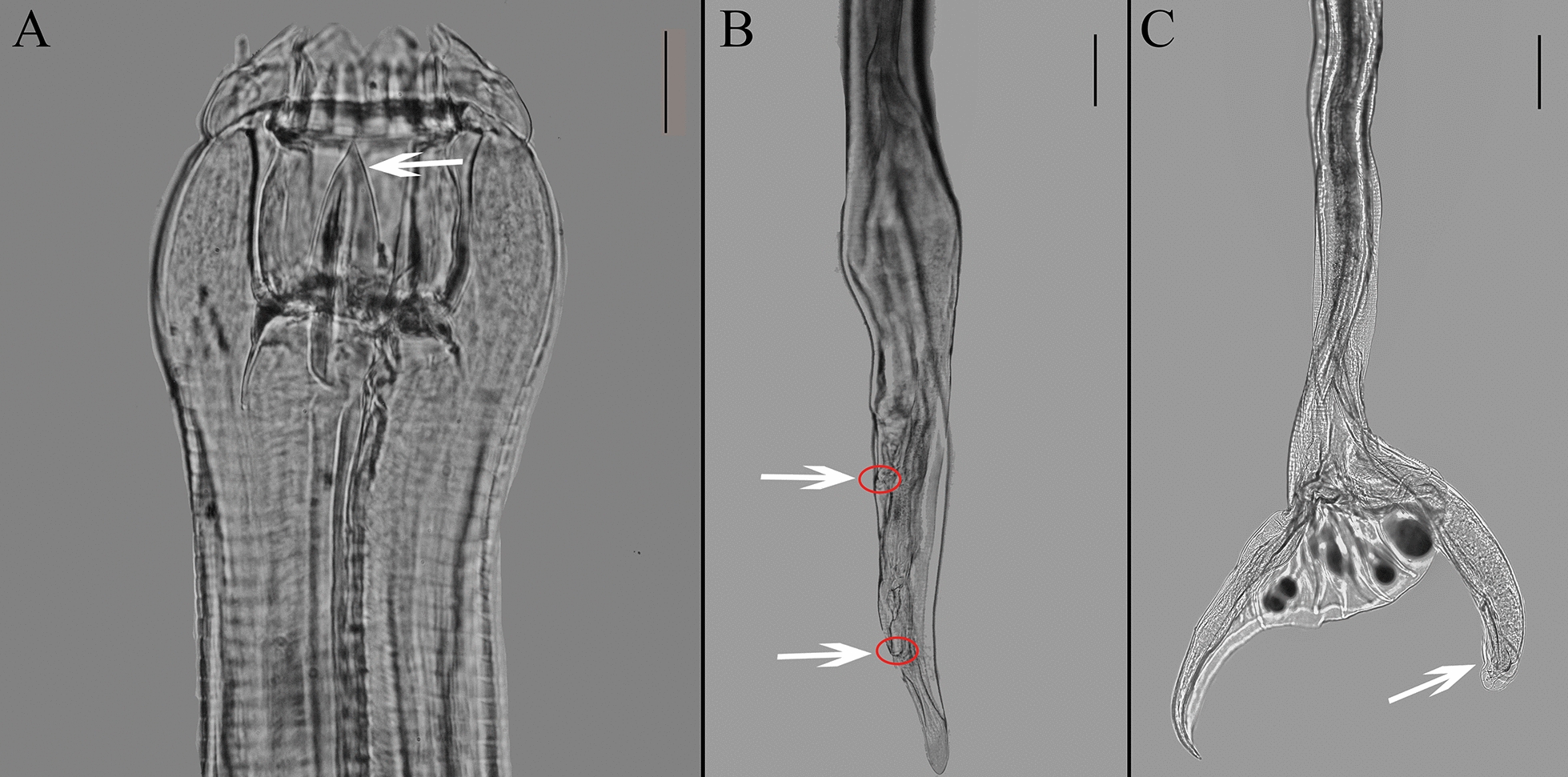
Light micrographs of *Bidentostomum ivashkini*. **A** Anterior part of body, lateral view. Arrow marks large dorsal esophageal tooth (scale bar: 50 μm). **B** Female tail, lateral view. Arrow at the anterior tip marks vulva. Arrow at the posterior tip marks anus (scale bar: 200 μm). **C** Male tail, lateral view. Genital cone, lateral view (arrow) (scale bar: 200 μm)

**Fig. 3:**
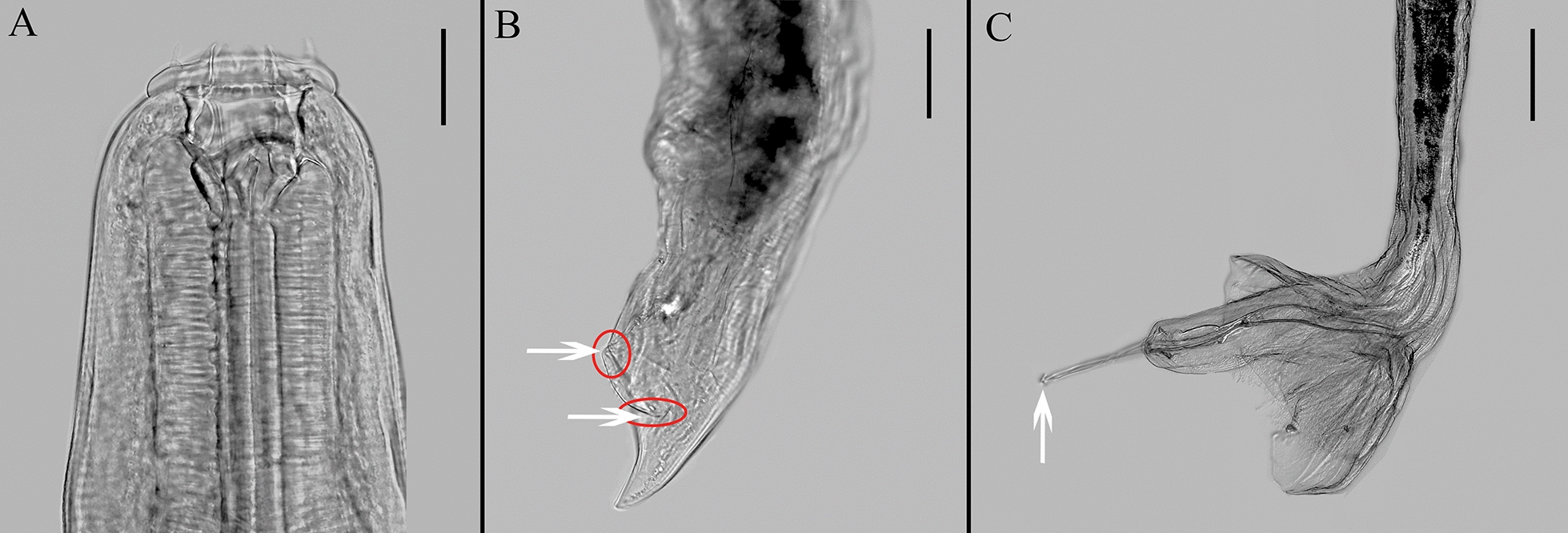
Differential interference micrographs of *Skrjabinodentus caragandicus*. **A** Anterior part of body, lateral view (scale bar: 50 μm). **B** Female tail, lateral view. Arrow at the anterior tip marks vulva. Arrow at the posterior tip marks anus (scale bar: 200 μm). **C** Male tail, lateral view. Spicule (arrow) (scale bar: 100 μm)

**Fig. 4:**
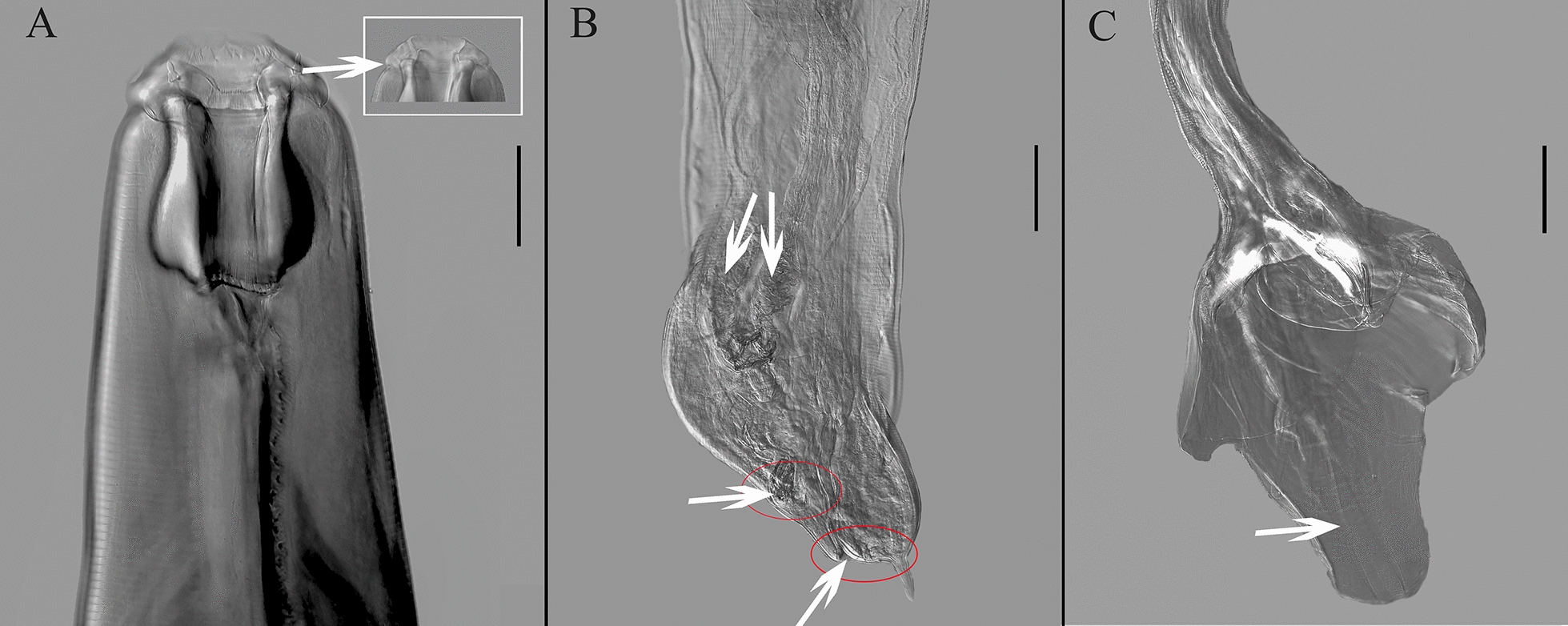
Differential interference micrographs of *Petrovinema skrjabini*. **A** Anterior part of body, lateral view. Arrow marks anterior part of head, dorsoventral view (scale bar: 100 μm). **B** Tail of female (scale bar: 200 μm). Arrows at the anterior tip mark vagina. The arrow (horizontal arrow) in the middle marks vulva. Arrow at the posterior tip marks anus. **C** Male tail, lateral view (scale bar: 200 μm)

For the first time to our knowledge, we employed differential interference contrast microscopy to capture lateral and ventral views of the tails of female and male specimens of *O. robustus*, *S. caragandicus* and * P. skrjabini* (Table 2). The primary morphological traits for females included reproductive organs such as the vulva (Figs. 1B, 3B,  4B), anus (Figs. 1B, 2B, 4B) and paired ovijectors (Fig 4B), revealing detailed structural features. Male identification traits included bursal structure, spicules (Fig. 3C) and genital cone. For instance, the genital cone of *S. caragandicus* is more developed and extends beyond the bursal edge compared to *O. robustus* (Figs. 1C, 3C). Additionally, the dorsal lobe of the bursa in the male tail (Fig. 4C) served as a distinguishing feature. A photograph of a female *B. ivaschkini*’s tail, captured under light microscopy, is included (Fig. 2B). The male tail of *B. ivaschkini* matched the light microscopy photograph provided by Bu [29] and did not necessitate further description.

**Table 2:** Detailed information of morphological characteristics of the four strongylid species

Measurement Item	*Oesophagodontus robustus*		*Bidentostomum ivashkini*		*Skrjabinodentus caragandicus*		*Petrovinema skrjabini*	
	Female	Male	Female	Male	Female	Male	Female	Male
Number (individuals)	5	3	6	2	8	8	8	8
Body length (mm)	19.0–23.0	14.2–15.4	9.0–24.0	7.6–9.1	8.3–10.9	7.9–9.6	13.5–16.8	12.1
Number of external leaf-crown leaves	18	18	8	8	8	8	28	28
Buccal capsule width (μm)	390.6	212.9–335.9	46.0–58.0	72.6	64.8	57.1	46.0–58.0	57.1
Buccal capsule depth (μm)	329.2	259.5	17.0–25.0	70.4	26.8	29.7	145.0–180.0	135.0–153.0
Length of vulva to tail tip (mm)	3.00–3.40	–	0.54–0.73	–	0.36–0.39	–	0.35–0.46	–
Length of anus to tail tip (mm)	0.63	–	0.22–0.29	–	0.16	–	0.18–0.28	–
Spicule length (mm)	–	0.60–0.71	–	0.88	–	1.02–1.08	–	0.62–0.77
Dorsal lobe of bursa length (mm)	–	0.33–0.36	–	0.75	–	0.43–0.46	–	0.56–0.66

The nuclear ITS2 sequences of the four species were individually compared with those available in the GenBank database using BLAST and NCBI. The molecular data for three strongylid species (*O. robustus*, *B. ivashkini* and *S. caragandicus*) aligned well with the morphological findings, exhibiting a high identity score of 98.44% and closely matching the respective accession number (Table 3). However, for *P. skrjabini*, there were no molecular data matches, and the BLAST results scored < 85% because of the absence of any molecular data for this species in the NCBI database. We obtained molecular data for *P. skrjabini* for the first time in this study.

**Table 3:** Comparative analysis of molecular data of BLAST results of four species of nematodes (*Oesophagodontus robustus, Bidentostomum ivashkini, Skrjabinodentus caragandicus, Petrovinema skrjabini*)

Species hypothesized	BLAST results in NCBI with accession numbers	References to the relevant publications	Identity score of the closest match accession number
*Oesophagodontus robustus*	Y08592.1 (*O. robustus*)	Hung et al. 2000	99.70%
*Bidentostomum ivashkini*	KP693440.1 (*B. ivashkini*)	Bu. 2016	99.7%
*Skrjabinodentus caragandicus*	KT984870.1 (*S. caragandicus*)	Zhang et al. 2016	98.44%
*Petrovinema skrjabini*	*——*		*——*

## Discussion

Parasitic infections have a broad transmission range and significantly impact wildlife [30]. As PH populations are being revitalized through animal reintroduction programs, understanding the potential risks associated with parasitic infections in these newly established populations is essential.

In this study, we made discoveries using both morphological and molecular techniques. We identified four strongylid species that use PH as a host. While previous studies have reported the presence of four strongylid species in *Equus caballus*, *E. asinus*, E. caballus * E. asinus and *E. burchelli*, their prevalence in PHs worldwide remains undocumented [8, 10, 31]. The species *O. robustus* and *B. ivaschkini* are rarely reported in equids and have only been recorded in China and Mongolia [8, 31]. Notably, the genus *Oesophagodontus* was first identified in PHs. *Skrjabinodentus caragandicus* has only been reported in China, specifically in a single case involving domestic donkeys in Henan Province. *Petrovinema skrjabini* is a distinct strongylid found less commonly than *P. poculatum* and is present in horses and donkeys [31].

Although Lichtenfels et al. [8] and Zhang and Kong [10] have described the morphological features of these parasites, their illustrations of male and female tails were hand drawn, introducing a level of subjectivity. For the first time to our knowledge, our study includes photographs of both male and female tails in the main text, which enhances the accuracy of classification and research. These photographs are instrumental in studying the sexual characteristics of the species. Future work should employ more advanced microscopic techniques, such as electron microscopy, to contribute effectively to PHs nematode research.

The molecular data for *P. skrjabini*'s ITS2 will be useful for accurately identifying this species in future studies. This data can also be employed to construct phylogenetic relationships between *P. skrjabini* and related strongylids, offering insights into their evolutionary relationships and taxonomic positions. Our study supplies critical evidence for future parasitic taxonomy and evolutionary research.

Horses are prone to symptoms such as depression, limb weakness, loss of appetite and severe anemia when parasitized by strongylid nematodes [32–35]. The high prevalence of strongylids in both locations underscores the importance of investigating the impact of these parasites on PH populations. Since parasitic infections are inevitable under both captive and wild conditions, understanding their epidemiology is crucial. Equid strongylid nematodes have been substantiated as generalist parasites within *Equus* [36]. In KNR, Przewalski's horses are living sympatrically with Mongolian wild asses (*Equus hemionus hemionus*), and the parasitic nematode species of these two animals can mutually transmit. These Mongolian wild asses freely traverse the border areas between Xinjiang and Inner Mongolia, making this region particularly interesting for parasitological research. Identifying new sources of infection in this area is crucial for understanding the distribution and impact of these nematode species in equine animals. Future research should focus on parasitic diseases and include long-term monitoring.

## Conclusions

This study reports the first documented instances, to our knowledge, of *O. robustus*, *B. ivaschkini*, *S. caragandicus* and *P. skrjabini* parasitizing *E. przewalskii*. Additionally, we acquired molecular data (ITS2) for *P. skrjabini*. These findings broaden the spectrum of parasitic nematode species known to infect PHs, thereby aiding further detailed research on the relationship between PHs and parasites.

### Supplementary Information

**Additional file 1: Table S1**. The list of strongylid species found in the Przewalski’s horses (*Equus ferus przewalskii*) in the in Xinjiang Uygur Autonomous Region wild horse Breeding Research Center (XRBRPH) and Kalamaili Nature Reserve (KNR) in China.

## Data Availability

All data generated or analyzed during this study are included in this published article.

## Abbreviations

PH: Przewalski’s horse (*Equus ferus przewalskii*)
BLAST: Basic Local Alignment Search Tool
