# Supplementary material for: First report of four rare strongylid species infecting endangered Przewalski’s horses (Equus ferus przewalskii) in Xinjiang, China
Source: Parasit Vectors. 2023 Oct 25;16:385. doi: 10.1186/s13071-023-05993-w (PMC10601325; doi:10.1186/s13071-023-05993-w)
Supplement: Supplementary file 1 — Additional file 1: Table S1. The list of strongylid species found in the Przewalski’s horses (Equus ferus przewalskii) in the in Xinjiang Uygur Autonomous Region wild horse Breeding Research Center (XRBRPH) and Kalamaili Nature Reserve (KNR) in China. [file 13071_2023_5993_MOESM1_ESM.docx]

**Additional file 1: Table S1.** The list of strongylid species found in the Przewalski’s horses (*Equus ferus przewalskii*) in the in Xinjiang Uygur Autonomous Region wild horse Breeding Research Center (XRBRPH) and Kalamaili Nature Reserve (KNR) in China.

| **Sub-family** | **Genus** | **Species** |
| --- | --- | --- |
| Strongylinae | *Strongylus* | *Strongylus equinus* Müller, 1780 |
| Strongylinae | *Strongylus* | *S. edentatus* (Looss, 1900) Railliet and Henry, 1909 |
| Strongylinae | *Strongylus* | *S. vulgaris* (Looss, 1900) Railliet and Henry, 1909 |
| Strongylinae | *Triodontophorus* | *Triodontophorus serratus* (Looss, 1900) Looss, 1902 |
| Strongylinae | *Oesophagodontus* | *Oesophagodontus robustus* (Giles, 1892) Railliet and Henry, 1902 |
| Strongylinae | *Bidentostomum* | *Bidentostomum ivaschkini* Tshoijo in Popova (1958) |
| Cyathostominae | *Cyathostomum* | *Cyathostomum catinatum* Looss, 1900 |
| Cyathostominae | *Cyathostomum* | *C. pateratum* (Yorke and Macfie, 1919) Cram, 1924 |
| Cyathostominae | *Coronocyclus* | *Coronocyclus coronatus* (Looss, 1900) Hartwich, 1986 |
| Cyathostominae | *Coronocyclus* | *C. labiatus* (Looss, 1902) Hartwich, 1986 |
| Cyathostominae | *Coronocyclus* | *C. labratus* (Looss, 1900) Hartwich, 1986 |
| Cyathostominae | *Cylicodontophorus* | *Cylicodontophorus bicoronatus* (Looss, 1900) Cram, 1924 |
| Cyathostominae | *Cylicocyclus* | *Cylicocyclus nassatus* (Looss, 1900) Chaves, 1930 |
| Cyathostominae | *Cylicocyclus* | *Cylicocyclus elongatus* (Looss, 1900) Chaves, 1930 |
| Cyathostominae | *Cylicocyclus* | *Cylicocyclus leptostomum* Kotlán, 1920 |
| Cyathostominae | *Cylicostephanus* | *Cylicostephanus goldi* (Boulenger, 1917) Lichtenfels, 1975 |
| Cyathostominae | *Cylicostephanus* | *Cylicostephanus minutus* (Yorke and Macfie, 1918) Cram, 1924 |
| Cyathostominae | *Cylicostephanus* | *C. calicatus* (Looss, 1900) Cram, 1924 |
| Cyathostominae | *Cylicostephanus* | *C. longibursatus* (Yorke and Macfie, 1918) Cram, 1924 |
| Cyathostominae | *Gyalocephalus* | *Gyalocephalus capitatus* Looss, 1900 |
| Cyathostominae | *Skrjabinodentus* | *Skrjabinodentus caragandicus* (Funikova, 1939) Tshoijo in Popova (1958) |
| Cyathostominae | *Petrovinema* | *Petrovinema skrjabini* (Ershov, 1930) Ershov, 1943 |
| Cyathostominae | *Parapoteriostomum* | *Parapoteriostomum euproctus* (Boulenger, 1917) Hartwich, 1986 |
